# Diffusion MRI Fiber Tractography and Benzodiazepine SPECT Imaging for Assessing Neural Damage to the Language Centers in an Elderly Patient after Successful Reperfusion Therapy

**DOI:** 10.3390/geriatrics9020030

**Published:** 2024-03-01

**Authors:** Tatsushi Mutoh, Yasuyuki Yoshida, Yasuko Tatewaki, Hongkun Chin, Ryota Tochinai, Junta Moroi, Tatsuya Ishikawa

**Affiliations:** 1 Department of Surgical Neurology, Research Institute for Brain and Blood Vessels, Akita Cerebrospinal and Cardiovascular Center, Akita 010-0874, Japan; 2 Department of Aging Research and Geriatric Medicine, Institute of Development, Aging and Cancer, Tohoku University, Aoba-ku, Sendai 980-8575, Japan; 3 Department of Veterinary Pathophysiology and Animal Health, Graduate School of Agricultural and Life Sciences, The University of Tokyo, Tokyo 113-8657, Japan

**Keywords:** acute ischemic stroke, post-stroke aphasia, benzodiazepine receptor imaging, diffusion tensor imaging, reperfusion therapy

## Abstract

Background: Intravenous thrombolysis and mechanical thrombectomy are the first-line reperfusion therapies for acute ischemic stroke. Here, we describe the utility of diffusion magnetic resonance imaging (MRI) fiber tractography and ^123^I-iomazenil benzodiazepine receptor single-photon emission computed tomography to estimate the prognosis of post-stroke aphasia after successful reperfusion therapy. Case report: An 81-year-old man was admitted to the hospital approximately 3.5 h after the onset of symptoms, including decreased consciousness, right hemiparesis, and aphasia. An MRI revealed acute cerebral infarction due to M1 segment occlusion. Intravenous alteplase thrombolysis followed by endovascular thrombectomy resulted in recanalization of the left middle cerebral artery territory. A subsequent MRI showed no new ischemic or hemorrhagic lesions. Although the patient’s motor hemiparesis gradually recovered, motor aphasia persisted. Diffusion MRI fiber tractography performed 2 weeks after admission revealed partial injury to the left arcuate fasciculus, indicated by lower fractional anisotropy values than on the contralateral side. A decreased benzodiazepine receptor density was also detected in the left perisylvian and temporoparietal cortices. The patient showed no clear signs of further improvement in the chronic stage post-stroke and was discharged to a nursing home after 3 months. Conclusions: The application of functional neuroimaging techniques to assess neuronal damage to the primary brain regions 2 weeks after reperfusion therapy for large-vessel occlusion may allow for an accurate prognosis of post-stroke aphasia. This may have a direct clinical implication for navigating subacute-to-chronic phases of rehabilitative care.

## 1. Introduction

Reperfusion therapy using intravenous thrombolysis and mechanical thrombectomy is a first-line treatment for acute ischemic stroke (AIS) due to large-vessel occlusion [1,2]. However, approximately half of patients do not achieve meaningful long-term functional improvement, despite technically successful recanalization of the occluded artery and reperfusion of the ischemic territory. Several epidemiological studies have proposed the concept of “futile reperfusion” and its association with many prognostic risk factors, including older age, female sex, a medical history of hypertension or diabetes, presence of cerebral microbleeds, higher systolic blood pressure or serum glucose level on admission, a higher National Institutes of Health Stroke Scale (NIHSS) score, the location of the occluded vessel, and procedure time intervals [3,4,5,6,7]. Furthermore, futile reperfusion is also associated with increased incidences of symptomatic intracranial hemorrhage and 90-day mortality [8].

Therefore, the development of novel, effective approaches to acute/subacute neuroprotection and chronic neurorestoration is expected for patients who still do not regain functional independence after successful reperfusion therapy. However, clinical trials that target neuronal activity critically involved in a specific phase and brain region have largely failed. An assessment of aphasia progression and recovery during the acute phase is particularly difficult in patients with no apparent cortical structural damage after enhancing the cerebral blood flow using conventional techniques, including magnetic resonance imaging (MRI) and perfusion scans such as enhanced computed tomography (CT).

It has been postulated that re-organization of the structure and function of the damaged brain via brain plasticity and improvement in the functional connection between regions are involved in restorative mechanisms during the recovery/chronic phase of a stroke [9]. Thus, we hypothesized that if we focus on identifying the intrinsic mechanism of neuronal activity during the transition period from neuroprotection to restoration (i.e., the late-acute-to-early-subacute stroke phase, approximately 2–3 weeks after onset), then it may help establish more personalized therapeutic strategies for neurorehabilitation.

In this report, we present a case demonstrating the utility of combined multimodal neuroimaging using diffusion MR fiber tractography and ^123^I-iomazenil benzodiazepine receptor single-photon emission computed tomography (IMZ-SPECT) for assessing neural damage related to prolonged aphasia after successful reperfusion therapy for large-vessel occlusion.

## 2. Case Presentation

**Figure 1:**
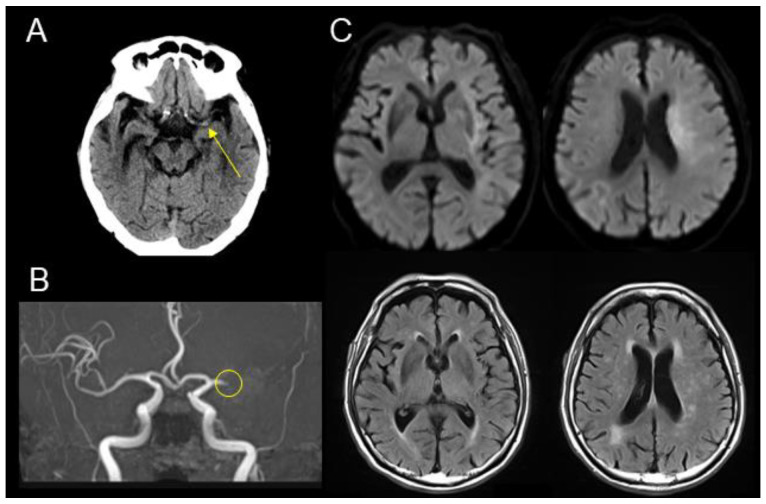
Preprocedural imaging examination upon admission. (**A**) Brain CT showed a high-density sign of the left middle cerebral artery (arrow). (**B**) Magnetic resonance angiography revealed a left M1 occlusion (circle). (**C**) Diffusion-weighted imaging (DWI) (upper raw) showed an ischemic area of the left insular cortex, internal capsule, basal ganglia, corona radiata, and hemispheric subcortical white matter (DWI-ASPECTS of 6 points). No apparent hyperintense signals were identified on fluid-attenuated inversion recovery (FLAIR) images (lower raw), suggestive of DWI–FLAIR mismatch.

An 81-year-old right-handed man with a medical history of type 2 diabetes mellitus was admitted by ambulance to the emergency room of our hospital with decreased consciousness and conjugate gaze palsy. The time last known well (TLKW) was approximately 3 h and 40 min prior to hospital arrival. Upon presentation, the patient revealed total aphasia, unilateral spatial neglect, and right-sided hemiparesis with Manual Muscle Testing (MMT) [10] grade 1 (flicker of contraction). The movement of the tongue and palate was not disturbed, and the electrocardiographic findings were normal. The patient’s NIHSS score was 22. A head CT revealed a hyperdense left middle cerebral artery (MCA) sign (Figure 1A). Additionally, diffusion-weighted imaging (DWI) of the brain, a structural MRI, and magnetic resonance angiography (MRA) revealed AIS with a DWI–FLAIR (fluid-attenuated inversion recovery) mismatch [11] due to left MCA occlusion (Figure 1B,C). The patient’s DWI-ASPECTS (Alberta Stroke Program Early CT Score) was seven.

Intravenous alteplase thrombolysis was performed immediately after the CT scan, 24 min after admission (before starting the MRI exam), followed by a mechanical thrombectomy 44 min after admission. The angiography showed a recanalization of the trunk and branches of the left MCA with modified treatment in cerebral infarction (mTICI) grade 3 at 131 min after admission (approximately 351 min after the TLKW) (Figure S1).

**Figure 2:**
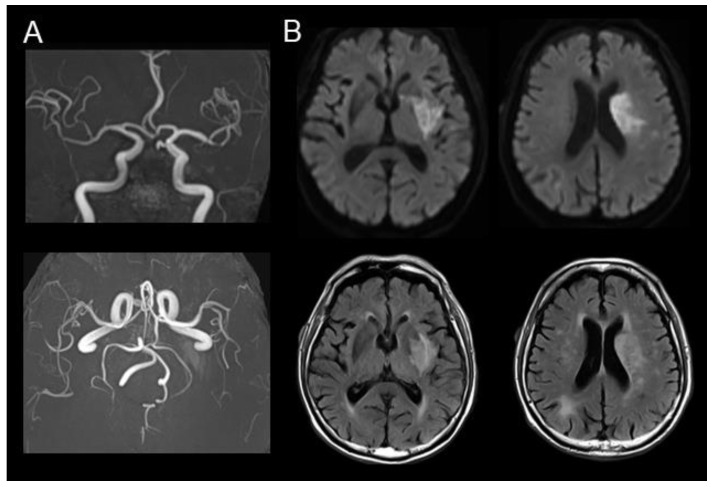
Postoperative imaging examination two days after reperfusion therapy. (**A**) Magnetic resonance angiography demonstrated successful recanalization of the left M1. (**B**) Both diffusion-weighted and fluid-attenuated inversion recovery images showed increased signals in the same area as those detected in initial MRI. Large parts of the frontotemporal gyri, including the primary sensorimotor and speech cortices, were spared on structural MRI.

After reperfusion therapy, the patient regained consciousness and exhibited symptoms of motor aphasia. A brain MRI showed no new ischemic or hemorrhagic lesions that did not have MRA-based evidence of early re-occlusion (Figure 2). The patient showed paroxysmal atrial fibrillation during 24 h Holter echocardiogram monitoring, and oral anticoagulant apixaban administration was started. Over the following week, his right limb muscle strength gradually recovered to MMT grade 4 (active movement against resistance but not to full strength); however, motor aphasia and buccofacial apraxia persisted, requiring a nasogastric tube for feeding and the prevention of aspiration pneumonia. A detailed mental assessment could not be performed because of the symptoms related to aphasia and apraxia.

**Figure 3:**
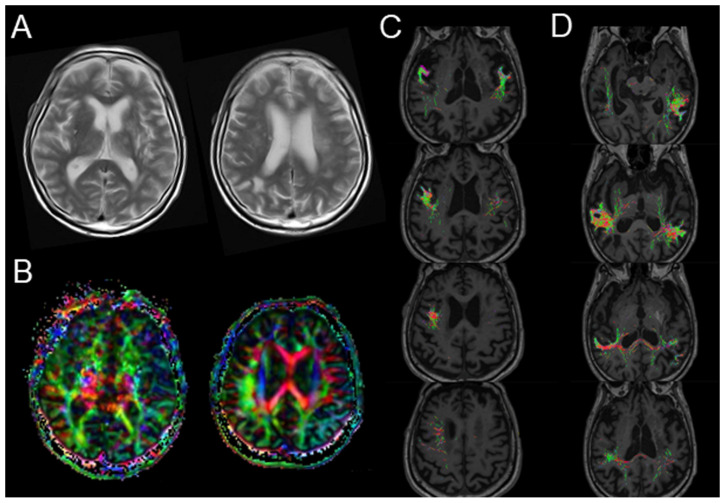
Postoperative MRI performed 2 weeks after reperfusion therapy. (**A**) T2-weighted brain MRI showed no new lesions. (**B**) Three-dimensional anisotropy contrast images showed darker areas in the left perisylvian cortex and lateral temporo-parietal lobe compared with those on the contralateral side. The fractional anisotropy map of the arcuate fasciculus using diffusion tensor imaging tractography-based anatomical region-of-interest seeding in Broca’s (**C**) or Wernicke’s area (**D**).

**Figure 4:**
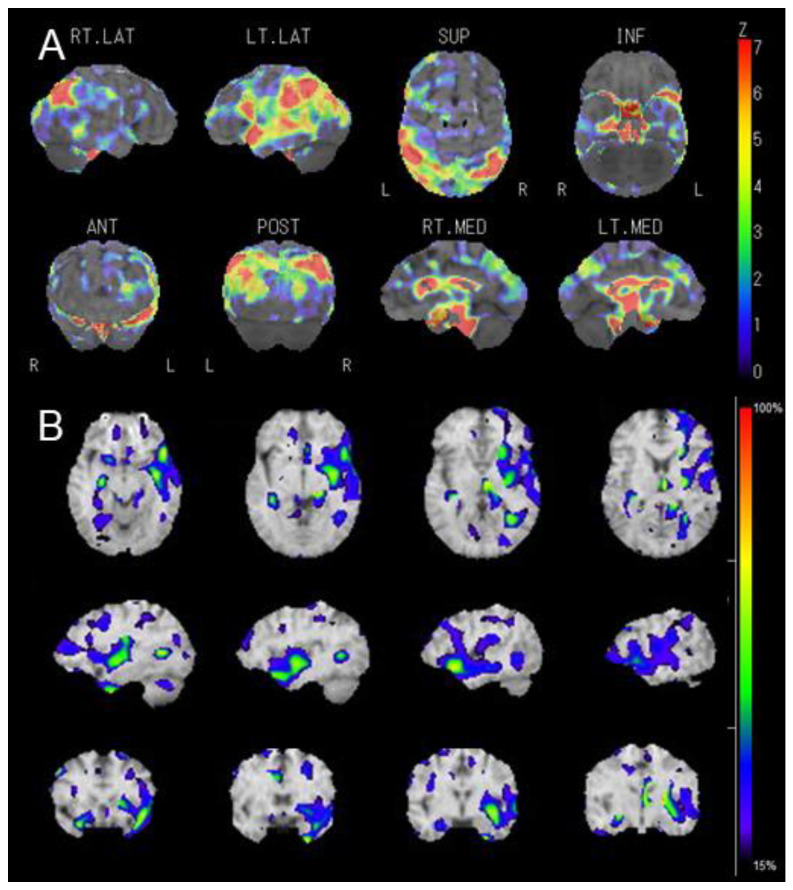
Postoperative IMZ-SPECT performed 2 weeks after reperfusion therapy. Statistical imaging analysis using three-dimensional stereotactic surface projection adjusted to global mean cerebral blood flow during the early phase (**A**) and asymmetry index during the late phase (**B**). See original images in Figure S2.

Diffusion MRI tractography performed 2 weeks after admission revealed partial injury to the left arcuate fasciculus, although no new lesions or atrophic changes in the cortical areas were observed in the structural MRI (Figure 3A,B). The images were preprocessed using tools and scripts provided by MRtrix33 [12]. Regarding the diffusion tensor imaging–fractional anisotropy (DTI-FA) map, the FA signals from the left Broca’s area projecting to the perisylvian cortex and superior longitudinal fasciculus could not be reconstructed, whereas those from Wernicke’s area were similar to those from the contralateral side (Figure 3C,D). The tensor metrics of decreased FA values and preserved mean diffusivity (MD) in the left projection from Broca’s area (Table S1) suggested a loss of neuronal integrity with no apparent microstructural changes in the arcuate fasciculus, even after successful recanalization and reperfusion. This was accompanied by decreased regional cerebral blood flow (CBF) and benzodiazepine receptor density in the left perisylvian and temporoparietal cortices, as evident in early and delayed IMZ-SPECT images (Figure 4 and Figure S2) [13].

**Figure 5:**
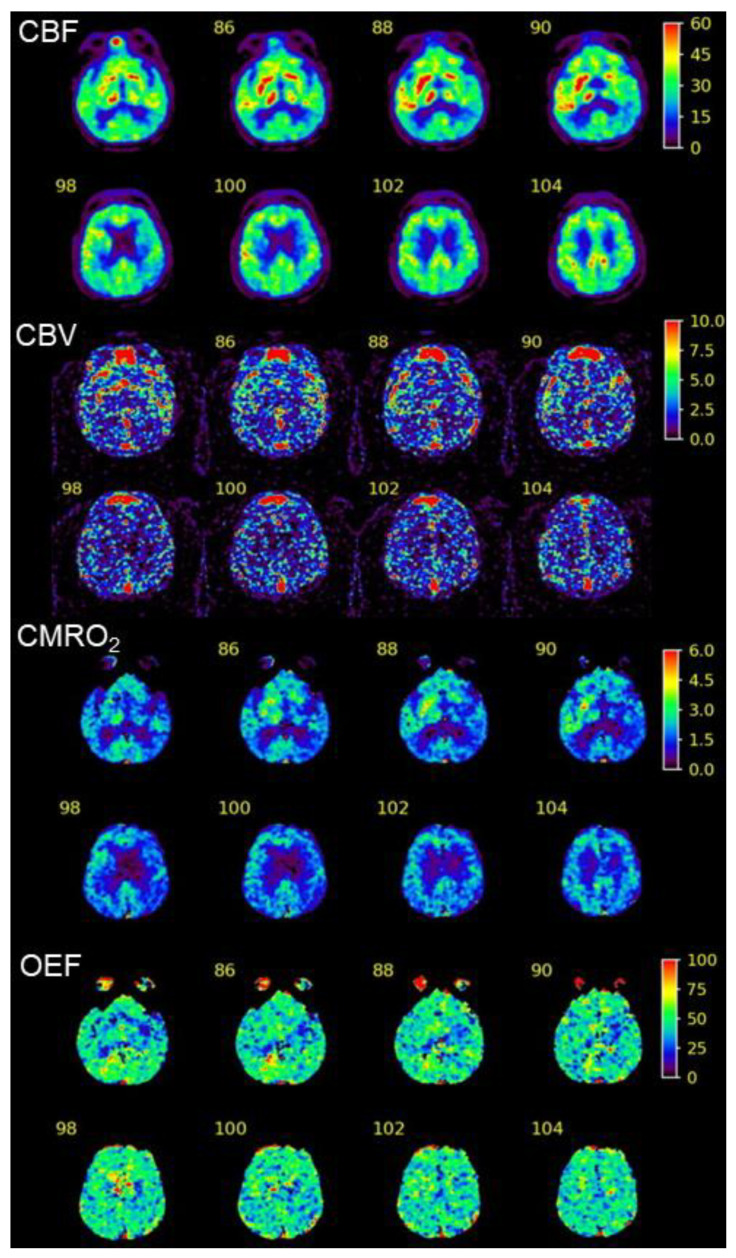
Hemodynamic maps from ^15^O-gas PET performed 2 weeks after reperfusion therapy. CBF, cerebral blood flow; CBV, cerebral blood volume; CMRO_2_, cerebral metabolic rate of oxygen; OEF, oxygen extraction fraction.

These findings corresponded with the areas of decreased CBF and cerebral metabolic rate of oxygen without an increased oxygen extraction fraction (i.e., a matched hypoperfusion–hypometabolism state) visualized through ^15^O-gas positron emission tomography (PET) (Figure 5) [14]. The reduced uptake of IMZ around the perisylvian cortex became more obvious and localized during the late phase, which may have indicated long-term neuronal damage to these areas (the language-relevant cortex and insula), compatible with residual neurological symptoms.

The patient exhibited no further neurological deterioration or radiological recurrence and was transferred to the rehabilitation department of the hospital 1 month after stroke onset with an NIHSS score of 16. Although his walking ability gradually improved up to therapist-assisted walking with a leg brace, no clear signs of recovery from aphasia and apraxia were observed in the chronic stage. With a modified Rankin scale score of four, the patient was discharged to a nursing home 3 months after admission.

## 3. Discussion

Aphasia is one of the most critical symptoms of AIS that may have a detrimental effect on activities in daily life, affecting approximately 30% of patients at stroke onset [15,16] and more than 10% in the chronic stage post-stroke [17]. Therefore, the prediction of the natural course of aphasia in stroke patients is of particular importance because it can provide useful information for planning specific rehabilitation strategies [18]. However, limited data exist regarding persistent aphasia and related neurological deficits after successful reperfusion therapy due to large-vessel occlusion [19,20,21]. This is the first report to visualize the neural damage to assess the prognosis for post-stroke aphasia in an elderly patient using MR fiber tractography and benzodiazepine receptor imaging.

The disruption of brain functional networks due to stroke is associated with impaired recovery from cognitive processes underlying language [22]. The left arcuate fasciculus is involved in various important neural tracts related to language function [23,24]. We estimated the white matter tract integrity and neuronal viability of the arcuate fasciculus using DTI fiber tracking and an assessment of the local distribution of central benzodiazepine receptor binding, respectively. MRI-based DTI and fiber tractography have several advantages in diagnosing neural functioning, which cannot be detected using conventional MRI. The serial scanning and analysis of asymmetry between the hemispheres using fiber tractography allow for an estimation of the regeneration, degeneration, or resolution of the perifocal edema of the lesion. Moreover, the relationships between clinical outcomes and neural tracts involved in language other than the arcuate fasciculus can be analyzed by comparing the neurological state of each patient. In a recent study using the diffusion parameter of microstructural integrity (MD index for gray matter), microstructural changes in the salvaged penumbra shortly following mechanical thrombectomy were associated with neurological and functional deficits found through the mRS after 3 months [20], supporting the potential clinical importance of analyzing DTI parameters in post-stroke patients.

The FA value represents the degree of directionality of water molecule movement in microstructures, such as axons, myelin, and microtubules, indicating connectivity. The integrity and size of the arcuate fasciculus was assessed using several DTI parameters (FA, MD, and fiber number) derived from fiber tractography [21]. In this patient, we observed a lack of FA map reconstruction and a lower FA value around the left perisylvian cortex and superior longitudinal fasciculus arising from Broca’s area. Recent data suggest that damage to the frontal regions, including Broca’s area, the insular cortex, and underlying white matter, is linked to poor spontaneous recovery from the subacute to the chronic stages post-stroke [25]. Conduction aphasia and comprehension deficits following stroke are reportedly associated with lower FA values for the left arcuate and superior longitudinal fasciculus [21]. Moreover, patients whose left arcuate fasciculus could not be reconstructed were found to have worse aphasia outcomes 6 months after stroke onset, irrespective of the preservation of white matter integrity in this region [18]. Taken together, residual microscopic neuronal damage after reperfusion therapy may have influenced the unfavorable outcomes in the present case.

The exact cortical origin and termination of the fibers assessed in this patient could not be determined using DTI or fiber tractography. Therefore, we speculated that a combination of functional neuroimaging techniques would be helpful to compensate for this limitation. Accordingly, we performed IMZ-SPECT and identified images that were positively correlated with CBF (early phase) and neural density to reflect cortical neural damage (late phase) [26,27,28,29]. Clinically and radiologically relevant data supporting neuronal damage associated with the arcuate fasciculus were clearly detected using late IMZ-SPECT. In addition, early IMZ-SPECT revealed broader areas of cerebral hypoperfusion than late imaging, extending to the left lateral temporoparietal cortex. Given the finding of a matched hypoperfusion state by ^15^O-gas PET, the imaging analyses suggested that this patient had a poor outcome due to aphasia and related symptoms and may still have been at risk of post-stroke cognitive impairment in strategic locations [30] due to the persistent hypoperfusion in the chronic phase of successful reperfusion therapy.

Numerous efforts have been conducted in searching for neuroprotective treatments to protect the brain against ischemia/reperfusion injury after reperfusion therapy [31]. During the acute/subacute stroke phase, spreading depolarization has been increasingly recognized as a key pathologic event that occurs spontaneously and contributes to secondary brain injury [32]. Measuring the content of potential biomarkers (e.g., S100b and neuron-specific enolase) that reflect neuronal damage and blood–brain barrier disruption could be useful for early clinical evaluations and outcomes after thrombolytic therapy [33,34]. However, specific and effective approaches that visualize the suppression of neuronal activity are not yet available. To improve functional recovery after AIS, earlier, non-invasive imaging would be beneficial for a precise diagnosis and treatment strategy in the chronic phase. For future directions, a new non-invasive approach such as MR fingerprinting and arterial spin labeling may provide a substitute for nuclear medicine imaging techniques for quantitative hemodynamic and structural imaging in stroke patients [35].

## 4. Conclusions

The present data demonstrate the utility of multimodal functional neuroimaging techniques to assess neuronal damage to the primary brain regions contributing to recovery or permanent disability from post-stroke aphasia. This may have a direct clinical implication for navigating subacute and chronic phases of rehabilitative care.

## Data Availability

The datasets used and/or analyzed during the current study are available from the corresponding author upon reasonable request.

## Supplementary Materials

The following supporting information can be downloaded at https://www.mdpi.com/article/10.3390/geriatrics9020030/s1: Figure S1: Mechanical thrombectomy of the left M1 occlusion; Figure S2: Original IMZ-SPECT images during the early phase (A) and late phase post-stroke (B); and Table S1: Diffusion parameters of the arcuate fasciculus.
