# Supplementary material for: Diffusion MRI Fiber Tractography and Benzodiazepine SPECT Imaging for Assessing Neural Damage to the Language Centers in an Elderly Patient after Successful Reperfusion Therapy
Source: Geriatrics (Basel). 2024 Mar 1;9(2):30. doi: 10.3390/geriatrics9020030 (PMC10961802; doi:10.3390/geriatrics9020030)
Supplement: Supplementary file 1 [file geriatrics-09-00030-s001.zip › Supplementary Figures S1-S2.pdf]

Supplementary data:

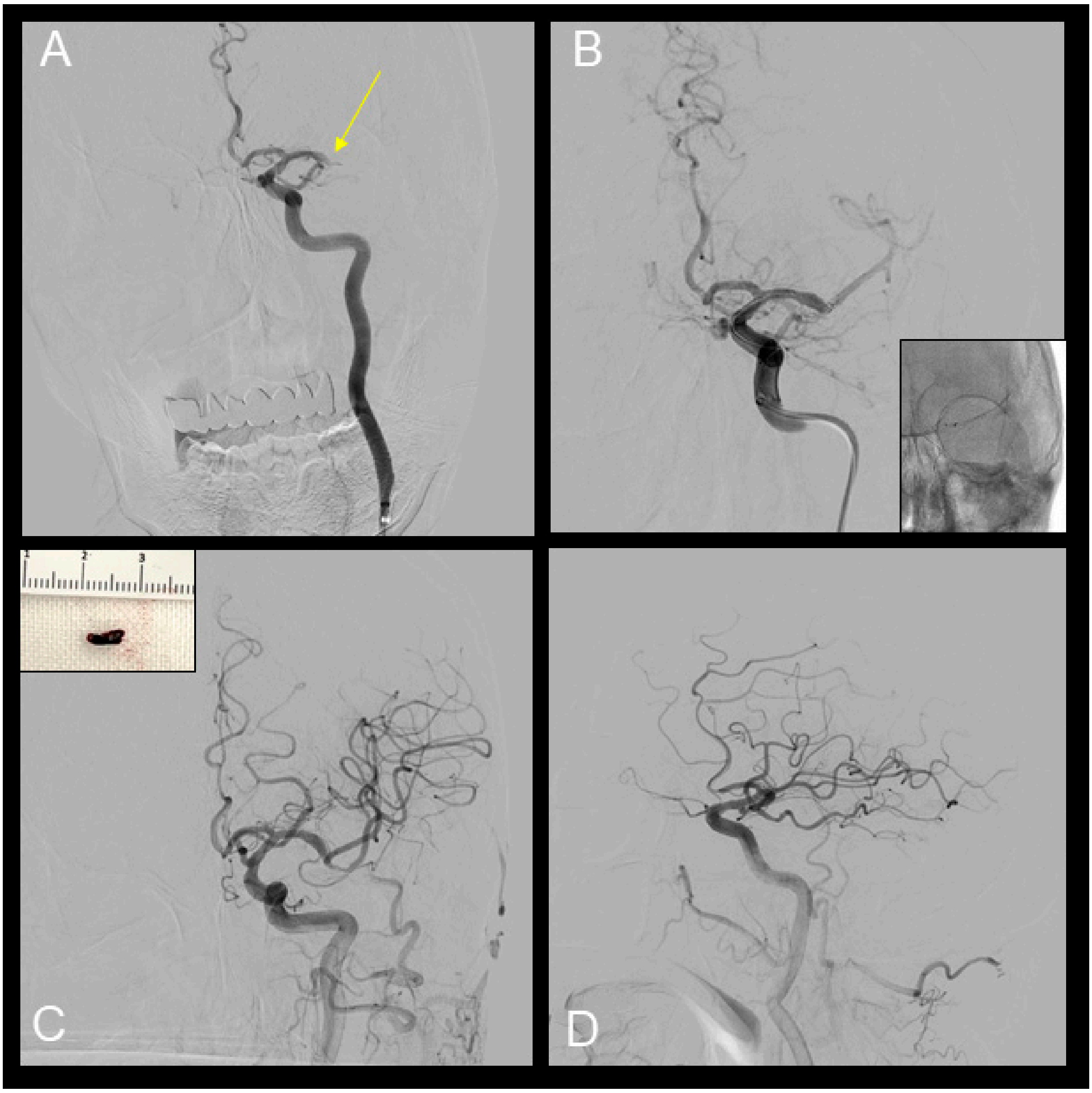

Figure S1. Mechanical thrombectomy of the left M1 occlusion. The Solitaire X stent device (4 mm x 40 mm; eV3, Irvine, CA, USA) and AXS Catalyst 6 aspiration catheter (Stryker Japan KK, Tokyo, Japan). (**A**) Left M1 occlusion (arrow). (**B)** The immediate flow restoration of one trunk of the left middle cerebral artery is achieved after deploying the stent retriever. (**C, D)** Final angiography shows full recanalization (modified thrombolysis in cerebral infarction grade 3) and the retrieved thrombus (**C,** Inset).

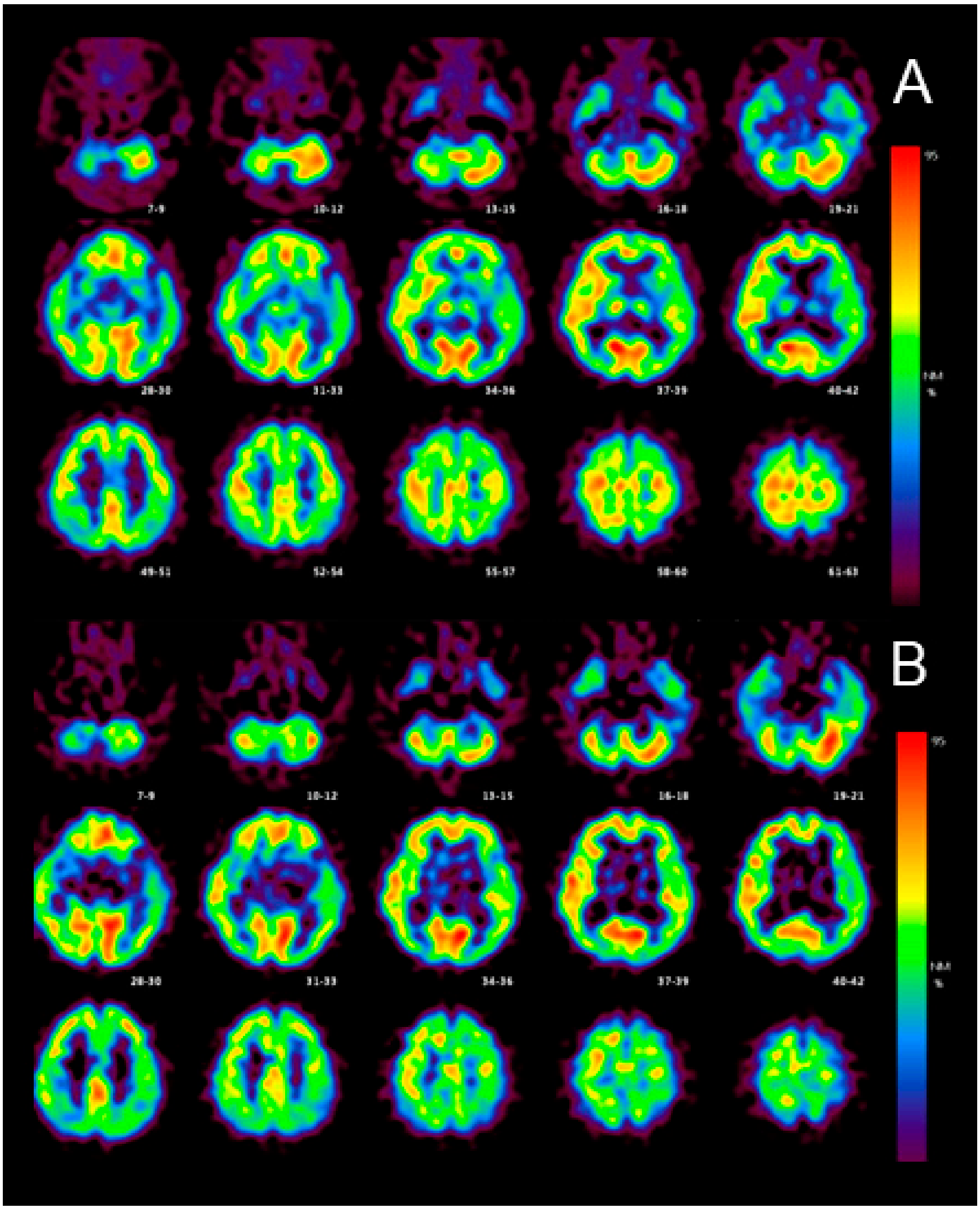

Figure S2. Original IMZ-SPECT images during the early phase (**A**) and late phase post-stroke (**B**).
