# Supplementary material for: Diffusion MRI Fiber Tractography and Benzodiazepine SPECT Imaging for Assessing Neural Damage to the Language Centers in an Elderly Patient after Successful Reperfusion Therapy
Source: Geriatrics (Basel). 2024 Mar 1;9(2):30. doi: 10.3390/geriatrics9020030 (PMC10961802; doi:10.3390/geriatrics9020030)
Supplement: Supplementary file 1 [file geriatrics-09-00030-s001.zip › Table S1.pdf]

Table S1. Diffusion parameters of the arcuate fasciculus.

	**Broca's area (L)**	**Broca's area (R)**	**Wernicke's area (L)**	**Wernicke's area (R)**
**FA**	**0.282815**	0.358052	0.319494	0.338662
**MD**	0.000874	0.000783	0.000894	0.000829
**FN**	1024	1024	1024	1024

Anatomical region-of-interest seeds were placed in the left (L) Broca's and Wernicke's areas. The values of the contralateral side (R) are presented as reference. FA, Fractional anisotropy; MD, Mean diffusivity; FN, Fiber number.
